# On the Microbe of Typhoid Fever

**Published:** 1882-12

**Authors:** Jno. A. Octerlony


### SELECTIONS.

### ON THE MICROBE OF TYPHOID FEVER.

During the last eight months, the author has been assiduously engaged in researches relating to the presence of a microbe in the living blood of typhoid fever. These researches were made upon a considerable number of preparations of normal and pathological blood before coagulation had occurred. After coagulation one often finds formations in the blood which perfectly resemble microbes, little rods of two or three segments or in the form of a chain. These formations probably consist of torn threads of fibrin. The methods used for the detection of microbes were always those of Weigert and of Koch. The method of coloring dried preparations was by means of aniline.

In the blood of persons suffering from typhoid fever there is often found a microbe, very small, and in very small numbers. It has the appearance of a small cylindrical rod, short (1.5 microm.) and fine; the extremities refract the light more strongly than the middle, which seems more transparent. This is probably the same organism which MM. Behier and Vulpian discovered in 1873.

Still more rarely are seen in typhoid blood other formations of short protoplasmic threads or ovoid grains. On rare occasions Dr. A. has established the presence of an enormous mass of microbes, but only for one or two days during the entire course of the disease.

There is no certainty of finding a larger number of microbes in severe cases than in abortive cases, in which latter class even he discovered a considerable number of them.

The microbe is most frequently encountered during the second and third week. He believes the pathogenic microbe must be sought at the bedside; there it is found in the patient's blood in the most pure state.

In order to obtain accurate results it is necessary to have perfectly pure seed for cultivation, or in inoculating animals. Authors have too much neglected researches of this kind. The microbe of the disease has been sought in the excreta, in the cadaver, in the soil, instead of commencing the search at the bedside.

It takes a good deal of time and trouble to find a sufficient number of the microbes of typhoid fever to serve for purposes of cultivation and inoculation.

It is well worth the trouble, however, for having once made the collection one does not have to wait long for results. In some infectious diseases the microbes are very easy to find, and soon it will be possible to determine the very day they are most abundant.

It was to be presumed that pathogenic microbes can be cultivated to perfection in normal blood, especially in the blood of an individual who has never been affected with the disease in question. It is now a well-established fact that normal blood is a fluid most favorable for the culture of the microbe of typhoid fever. Dr. A. cultivated this microbe, taken on the eleventh day from one of his own cases, in a drop inclosed between two glasses, one of which was concave. Thus he observed day by day the development of the micro-organism for three months. From this drop was produced a series of generations. With the microbe of the second generation he was able to successfully inoculate a dog. The result was quite interesting. The animal was hardly sick at all, but on the fifteenth day Peyer's patches were found swollen, and contained characteristic microbes. Although his researches have not yet reached completion, they seem to justify him in expressing the following opinions:

The microbe of typhoid fever is only accidentally present in the blood. It exists principally in the wall of the intestine, and ordinarily enters the blood only in small numbers. It is presumed that if more are found they are thrombi of microbes which having been detached from their places, circulate in the blood, and are broken up.

Six forms of the microbe are known at present:

1. Little rods found in the blood.

2. Very fine delicate threads, which the author has sometimes seen projecting from the above, the rod being then the spore of the micro-organism.

3. A mycelium so fine that the filamen of the genus baccillus are veritable giants in comparison with it. Under culture the form is found both in the blood and in the intestine. It is probably the body which Mr. Klebs has also discovered.

4. Zoöglea of very fine granules, very distinct and of uniform size, often in intimate relation with the fine threads already described. They have been found in the intestines, and under culture, and have been described by Klebs, Klein, and others.

5. Irregular masses of granules. These granules vary in size. They are ovoid, of indistinct contour. They have been perfectly described by M. Eberth, and have also been observed by other authors.

6. *Protoplasic masses*, more or less granular. They form very abundantly in blood under cultivation, and develop round or ovoid granules. Red blood-cells may be found for months inclosed in these masses. It may have been this form which M. Eichhorst observed in living blood, and perhaps some of the large cell formations found in the viscera may belong to it.

The microbe of typhoid can no longer be classed as of the genus bacillus, micrococcus, or bacterium. Dr. A. considers the following to be the successive phases of its development: The spore throws out a filament. Several filaments form either a network of filaments—a mycelium—or thick bundles of filaments—a filamentous zoöglea.

If spores develop in the filaments, the filamentous zoöglea may become transformed into a finely granular zoöglea.

The details of the mode of formation and development of the protoplasmic masses and of the granular masses the author has not yet been able to determine.—*Jno. A. Octerlony in Louisville Med. News.*

